# Adaptive designs in clinical trials: why use them, and how to run and report them

**DOI:** 10.1186/s12916-018-1017-7

**Published:** 2018-02-28

**Authors:** Philip Pallmann, Alun W. Bedding, Babak Choodari-Oskooei, Munyaradzi Dimairo, Laura Flight, Lisa V. Hampson, Jane Holmes, Adrian P. Mander, Lang’o Odondi, Matthew R. Sydes, Sofía S. Villar, James M. S. Wason, Christopher J. Weir, Graham M. Wheeler, Christina Yap, Thomas Jaki

**Affiliations:** 1 Department of Mathematics & Statistics, Lancaster University, Lancaster, LA1 4YF UK; 2 Roche Products Ltd, Welwyn Garden City, UK; 3 MRC Clinical Trials Unit at UCL, Institute of Clinical Trials and Methodology, University College London, London, UK; 4 Clinical Trials Research Unit, University of Sheffield, Sheffield, UK; 5 Medical Statistics Group, University of Sheffield, Sheffield, UK; 6 Statistical Innovation Group, Advanced Analytics Centre, AstraZeneca, Cambridge, UK; 7 Centre for Statistics in Medicine, University of Oxford, Oxford, UK; 8 MRC Biostatistics Unit, University of Cambridge, Cambridge, UK; 9 Institute of Health and Society, Newcastle University, Newcastle, UK; 10 Usher Institute of Population Health Sciences and Informatics, University of Edinburgh, Edinburgh, UK; 11 Cancer Research UK & UCL Cancer Trials Centre, University College London, London, UK; 12 Cancer Research UK Clinical Trials Unit, University of Birmingham, Birmingham, UK

**Keywords:** Statistical methods, Adaptive design, Flexible design, Interim analysis, Design modification, Seamless design

## Abstract

Adaptive designs can make clinical trials more flexible by utilising results accumulating in the trial to modify the trial’s course in accordance with pre-specified rules. Trials with an adaptive design are often more efficient, informative and ethical than trials with a traditional fixed design since they often make better use of resources such as time and money, and might require fewer participants. Adaptive designs can be applied across all phases of clinical research, from early-phase dose escalation to confirmatory trials. The pace of the uptake of adaptive designs in clinical research, however, has remained well behind that of the statistical literature introducing new methods and highlighting their potential advantages. We speculate that one factor contributing to this is that the full range of adaptations available to trial designs, as well as their goals, advantages and limitations, remains unfamiliar to many parts of the clinical community. Additionally, the term adaptive design has been misleadingly used as an all-encompassing label to refer to certain methods that could be deemed controversial or that have been inadequately implemented.

We believe that even if the planning and analysis of a trial is undertaken by an expert statistician, it is essential that the investigators understand the implications of using an adaptive design, for example, what the practical challenges are, what can (and cannot) be inferred from the results of such a trial, and how to report and communicate the results. This tutorial paper provides guidance on key aspects of adaptive designs that are relevant to clinical triallists. We explain the basic rationale behind adaptive designs, clarify ambiguous terminology and summarise the utility and pitfalls of adaptive designs. We discuss practical aspects around funding, ethical approval, treatment supply and communication with stakeholders and trial participants. Our focus, however, is on the interpretation and reporting of results from adaptive design trials, which we consider vital for anyone involved in medical research. We emphasise the general principles of transparency and reproducibility and suggest how best to put them into practice.

## Why, what and when to adapt in clinical trials

Traditionally, clinical trials have been run in three steps [1]:

- The trial is designed.
- The trial is conducted as prescribed by the design.
- Once the data are ready, they are analysed according to a pre-specified analysis plan.

**Fig. 1:**
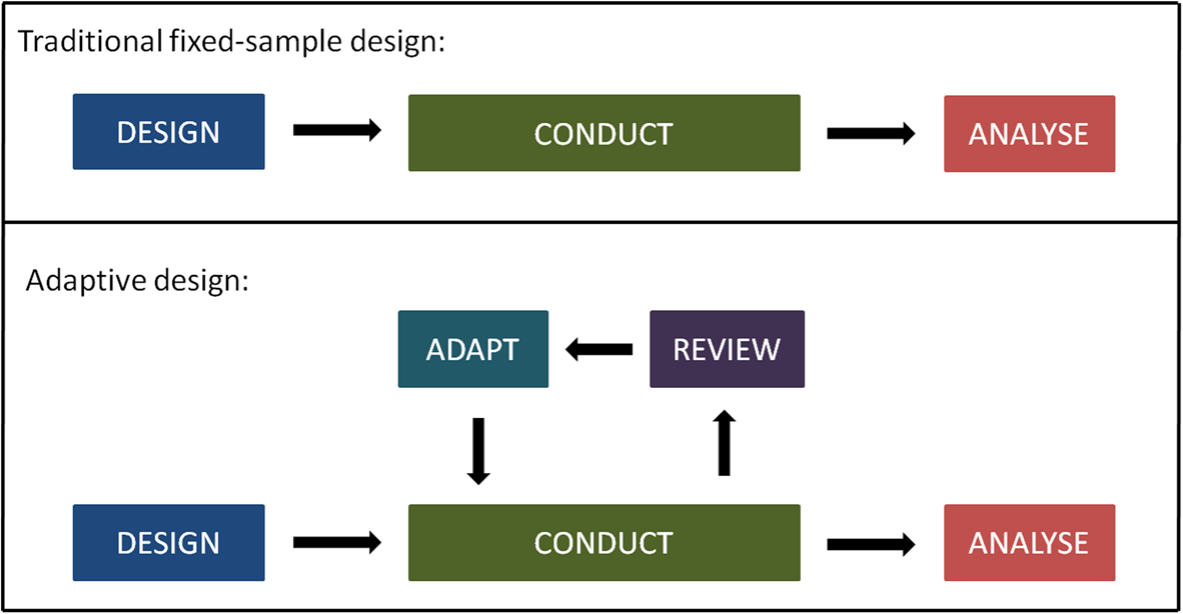
Schematic of a traditional clinical trial design with fixed sample size, and an adaptive design with pre-specified review(s) and adaptation(s)

This practice is straightforward, but clearly inflexible as it does not include options for changes that may become desirable or necessary during the course of the trial. Adaptive designs (ADs) provide an alternative. They have been described as ‘planning to be flexible’ [2], ‘driving with one’s eyes open’ [3] or ‘taking out insurance’ against assumptions [4]. They add a review–adapt loop to the linear design–conduct–analysis sequence (Fig. 1). Scheduled interim looks at the data are allowed while the trial is ongoing, and pre-specified changes to the trial’s course can be made based on analyses of accumulating data, whilst maintaining the validity and integrity of the trial. Such a priori planned adaptations are fundamentally different from unplanned ad hoc modifications, which are common in traditional trials (e.g. alterations to the eligibility criteria).

Pre-planned changes that an AD may permit include, but are not limited to [5]:

- refining the sample size
- abandoning treatments or doses
- changing the allocation ratio of patients to trial arms
- identifying patients most likely to benefit and focusing recruitment efforts on them
- stopping the whole trial at an early stage for success or lack of efficacy.

Table 1 lists some well-recognised adaptations and examples of their use. Note that multiple adaptations may be used in a single trial, e.g. a group-sequential design may also feature mid-course sample size re-estimation and/or adaptive randomisation [6], and many multi-arm multi-stage (MAMS) designs are inherently seamless [7]. ADs can improve trials across all phases of clinical development, and seamless designs allow for a more rapid transition between phases I and II [8, 9] or phases II and III [10, 11].

**Table 1:** Overview of adaptive designs with examples of trials that employed these methods

Design	Idea	Examples
Continual reassessment method	Model-based dose escalation to estimate the maximum tolerated dose	TRAFIC [136], Viola [137], RomiCar [138]
Group-sequential	Include options to stop the trial early for safety, futility or efficacy	DEVELOP-UK [139]
Sample size re-estimation	Adjust sample size to ensure the desired power	DEVELOP-UK [139]
Multi-arm multi-stage	Explore multiple treatments, doses, durations or combinations with options to ‘drop losers’ or ‘select winners’ early	TAILoR [31], STAMPEDE [67, 140], COMPARE [141], 18-F PET study [142]
Population enrichment	Narrow down recruitment to patients more likely to benefit (most) from the treatment	Rizatriptan study [143, 144]
Biomarker-adaptive	Incorporate information from or adapt on biomarkers	FOCUS4 [145], DILfrequency [146]; examples in [147, 148]
Adaptive randomisation	Shift allocation ratio towards more promising or informative treatment(s)	DexFEM [149]; case studies in [150, 151]
Adaptive dose-ranging	Shift allocation ratio towards more promising or informative dose(s)	DILfrequency [146]
Seamless phase I/II	Combine safety and activity assessment into one trial	MK-0572 [152], Matchpoint [153, 154]
Seamless phase II/III	Combine selection and confirmatory stages into one trial	Case studies in [133]

The defining characteristic of all ADs is that results from interim data analyses are used to modify the ongoing trial, without undermining its integrity or validity [12]. Preserving the integrity and validity is crucial. In an AD, data are repeatedly examined. Thus, we need to make sure they are collected, analysed and stored correctly and in accordance with good clinical practice at every stage. Integrity means ensuring that trial data and processes have not been compromised, e.g. minimising information leakage at the interim analyses [13]. Validity implies there is an assurance that the trial answers the original research questions appropriately, e.g. by using methods that provide accurate estimates of treatment effects [14] and correct *p* values [15–17] and confidence intervals (CIs) for the treatment comparisons [18, 19]. All these issues will be discussed in detail in subsequent sections.

The flexibility to make mid-course adaptations to a trial is not a virtue in itself but rather a gateway to more efficient trials [20] that should also be more appealing from a patient’s perspective in comparison to non-ADs because:

- Recruitment to futile treatment arms may stop early.
- Fewer patients may be randomised to a less promising treatment or dose.
- On average, fewer patients may be required overall to ensure the same high chance of getting the right answer.
- An underpowered trial, which would mean a waste of resources, may be prevented.
- A better understanding of the dose–response or dose–toxicity relationship may be achieved, thus, facilitating the identification of a safe and effective dose to use clinically.
- The patient population most likely to benefit from a treatment may be identified.
- Treatment effects may be estimated with greater precision, which reduces uncertainty about what the better treatment is.
- A definitive conclusion may be reached earlier so that novel effective medicines can be accessed sooner by the wider patient population who did not participate in the trial.

ADs have been available for more than 25 years [21], but despite their clear benefits in many situations, they are still far from established in practice (with the notable exception of group-sequential methods, which many people would not think to recognise as being adaptive) for a variety of reasons. Well-documented barriers [22–29] include lack of expertise or experience, worries of how funders and regulators may view ADs, or indeed more fundamental practical challenges and limitations specific to certain types of ADs.

We believe that another major reason why clinical investigators are seldom inclined to adopt ADs is that there is a lack of clarity about:

- when they are applicable
- what they can (and cannot) accomplish
- what their practical implications are
- how their results should be interpreted and reported.

To overcome these barriers, we discuss in this paper some practical obstacles to implementing ADs and how to clear them, and we make recommendations for interpreting and communicating the findings of an AD trial. We start by illustrating the benefits of ADs with three successful examples from real clinical trials.

## Case studies: benefits of adaptive designs

### A trial with blinded sample size re-estimation

Combination Assessment of Ranolazine in Stable Angina (CARISA) was a multi-centre randomised double-blind trial to investigate the effect of ranolazine on the exercising capacity of patients with severe chronic angina [30]. Participants were randomly assigned to one of three arms: twice daily placebo or 750 mg or 1000 mg of ranolazine given over 12 weeks, in combination with standard doses of either atenolol, amlodipine or diltiazem at the discretion of the treating physician. The primary endpoint was treadmill exercise duration at trough, i.e. 12 hours after dosing. The sample size necessary to achieve 90% power was calculated as 462, and expanded to 577 to account for potential dropouts.

After 231 patients had been randomised and followed up for 12 weeks, the investigators undertook a planned blinded sample size re-estimation. This was done to maintain the trial power at 90% even if assumptions underlying the initial sample size calculation were wrong. The standard deviation of the primary endpoint turned out to be considerably higher than planned for, so the recruitment target was increased by 40% to 810. The adaptation prevented an underpowered trial, and as it was conducted in a blinded fashion, it did not increase the type I error rate. Eventually, a total of 823 patients were randomised in CARISA. The trial met the primary endpoint and could claim a significant improvement in exercise duration for both ranolazine doses.

### A multi-arm multi-stage trial

Telmisartan and Insulin Resistance in HIV (TAILoR) was a phase II dose-ranging multi-centre randomised open-label trial investigating the potential of telmisartan to reduce insulin resistance in HIV patients on combination antiretroviral therapy [31]. It used a MAMS design [32] with one interim analysis to assess the activity of three telmisartan doses (20, 40 or 80 mg daily) against control, with equal randomisation between the three active dose arms and the control arm. The primary endpoint was the 24-week change in insulin resistance (as measured by a validated surrogate marker) versus baseline.

The interim analysis was conducted when results were available for half of the planned maximum of 336 patients. The two lowest dose arms were stopped for futility, whereas the 80 mg arm, which showed promising results at interim, was continued along with the control. Thus, the MAMS design allowed the investigation of multiple telmisartan doses but recruitment to inferior dose arms could be stopped early to focus on the most promising dose.

### An adaptive randomisation trial

Giles et al. conducted a randomised trial investigating three induction therapies for previously untreated, adverse karyotype, acute myeloid leukaemia in elderly patients [33]. Their goal was to compare the standard combination regimen of idarubicin and ara-C (IA) against two experimental combination regimens involving troxacitabine and either idarubicin or ara-C (TI and TA, respectively). The primary endpoint was complete remission without any non-haematological grade 4 toxicities by 50 days. The trial began with equal randomisation to the three arms but then used a response-adaptive randomisation (RAR) scheme that allowed changes to the randomisation probabilities, depending on observed outcomes: shifting the randomisation probabilities in favour of arms that showed promise during the course of the trial or stopping poorly performing arms altogether (i.e. effectively reducing their randomisation probability to zero). The probability of randomising to IA (the standard) was held constant at 1/3 as long as all three arms remained part of the trial. The RAR design was motivated by the desire to reduce the number of patients randomised to inferior treatment arms.

**Fig. 2:**
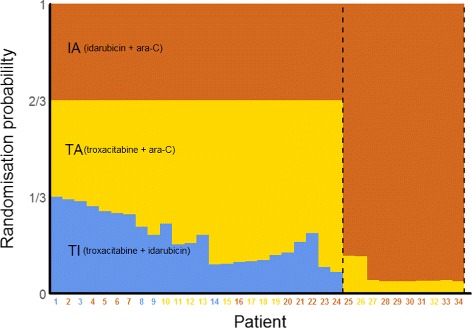
Overview of the troxacitabine trial using a response-adaptive randomisation design. The probabilities shown are those at the time the patient on the *x*-axis was randomised. Coloured numbers indicate the arms to which the patients were randomised

After 24 patients had been randomised, the probability of randomising to TI was just over 7%, so recruitment to this arm was terminated and the randomisation probabilities for IA and TA recalculated (Fig. 2). The trial was eventually stopped after 34 patients, when the probability of randomising to TA had dropped to 4%. The final success rates were 10/18 (56%) for IA, 3/11 (27%) for TA, and 0/5 (0%) for TI. Due to the RAR design, more than half of the patients (18/34) were treated with the standard of care (IA), which was the best of the three treatments on the basis of the observed outcome data, and the trial could be stopped after 34 patients, which was less than half of the planned maximum of 75. On the other hand, the randomisation probabilities were highly imbalanced in favour of the control arm towards the end, suggesting that recruitment to this trial could have been stopped even earlier (e.g. after patient 26).

## Practical aspects

As illustrated by these examples, ADs can bring about major benefits, such as shortening trial duration or obtaining more precise conclusions, but typically at the price of being more complex than traditional fixed designs. In this section, we briefly highlight five key areas where additional thought and discussions are necessary when planning to use an AD. Considering these aspects is vital for clinical investigators, even if they have a statistician to design and analyse the trial. The advice we give here is largely based on our own experiences with ADs in the UK public sector.

### Obtaining funding

Before a study can begin, funding to conduct it must be obtained. The first step is to convince the decision-making body that the design is appropriate (in addition to showing scientific merits and potential, as with any other study). This is sometimes more difficult with ADs than for traditional trial designs, as the decision makers might not be as familiar with the methods proposed, and committees can tend towards conservative decisions. To overcome this, it is helpful to ensure that the design is explained in non-technical terms while its advantages over (non-adaptive) alternatives and its limitations are highlighted. On occasion, it might also be helpful to involve a statistician with experience of ADs, either by recommending the expert to be a reviewer of the proposal or by including an independent assessment report when submitting the case.

Other challenges related to funding are more specific to the public sector, where staff are often employed for a specific study. Questions, such as ‘How will the time for developing the design be funded?’ and ‘What happens if the study stops early?’ need to be considered. In our experience, funders are often supportive of ADs and therefore, tend to be flexible in their arrangements, although decisions seem to be on a case-by-case basis. Funders frequently approve of top-up funding to increase the sample size based on promising interim results [34, 35], especially if there is a cap on the maximum sample size [36].

To overcome the issue of funding the time to prepare the application, we have experience of funders agreeing to cover these costs retrospectively (e.g. [37]). Some have also launched funding calls specifically to support the work-up of a trial application, e.g. the Joint Global Health trials scheme [38], which awards trial development grants, or the Planning Grant Program (R34) of the National Institutes of Health [39].

### Communicating the design to trial stakeholders

Once funding has been secured, one of the next challenges is to obtain ethics approval for the study. While this step is fairly painless in most cases, we have had experiences where further questions about the AD were raised, mostly around whether the design makes sense more broadly, suggesting unfamiliarity with AD methods overall. These clarifications were easily answered, although in one instance we had to obtain a letter from an independent statistical expert to confirm the appropriateness of the design. In our experience, communications with other stakeholders, such as independent data monitoring committees (IDMCs) and regulators, have been straightforward and at most required a teleconference to clarify design aspects. Explaining simulation results to stakeholders will help to increase their appreciation of the benefits and risks of any particular design, as will walking them through individual simulated trials, highlighting common features of data sets associated with particular adaptations.

The major regulatory agencies for Europe and the US have recently issued detailed guidelines on ADs [40–42]. They tend to be well-disposed towards AD trials, especially when the design is properly justified and concerns about type I error rate control and bias are addressed [43, 44]. We will expand on these aspects in subsequent sections.

### Communicating the design to trial participants

Being clear about the design of the study is a key requirement when recruiting patients, which in practice will be done by staff of the participating sites. While, in general, the same principles apply as for traditional designs, the nature of ADs makes it necessary to allow for the specified adaptations. Therefore, it is good practice to prepare patient information sheets and similar information for all possible adaptations at the start of the study. For example, for a multi-arm treatment selection trial where recruitment to all but one of the active treatment arms is terminated at an interim analysis, separate patient information sheets should be prepared for the first stage of the study (where patients can be randomised to control or any active treatment), and for the second stage, there should be separate sheets for each active versus control arm.

### IDMC and trial steering committee roles

Reviewing observed data at each interim analysis requires careful thought to avoid introducing bias into the trial. For treatment-masked (blinded) studies that allow changes that may reveal—implicitly or explicitly—some information about the effectiveness of the treatments (e.g. stopping arms or changing allocation ratios) it is important to keep investigators and other people with a vested interest in the study blinded wherever possible to ensure its integrity. For example, they should not see any unblinded results for specific arms during the study to prevent ad hoc decisions being made about discontinuing arms or changing allocation ratios on the basis of accrued data. When stopping recruitment to one or more treatment arms, it is necessary to reveal that they have been discontinued and consequently hard to conceal the identity of the discontinued arm(s), as e.g. patient information sheets have to be updated.

In practice, it is advisable to instruct a (non-blind) IDMC to review interim data analyses and make recommendations to a (blind) trial steering committee (TSC) with independent membership about how the trial should proceed [45–51], whether that means implementing the AD as planned or, if there are serious safety issues, proposing an unplanned design modification or stopping [41]. The TSC, whose main role is to oversee the trial [52–54], must approve any ad hoc modifications (which may include the non-implementation of planned adaptations) suggested by the IDMC. However, their permission is not required for the implementation of any planned adaptations that are triggered by observed interim data, as these adaptations are part of the initial trial design that was agreed upon. In some cases though, adaptation rules may be specified as non-binding (e.g. futility stopping criteria in group-sequential trials) and therefore, inevitably require the TSC to make a decision on how to proceed.

To avoid ambiguity, all adaptation rules should be defined clearly in the protocol as well as in the IDMC and TSC charters and agreed upon between these committees and the trial team before the trial begins. The sponsor should ensure that the IDMC and TSC have members with all the skills needed to implement the AD and set up firewalls to avoid undue disclosure of sensitive information, e.g. to the trial team [55].

### Running the trial

Our final set of practical challenges relates to running the study. Once again, many aspects will be similar to traditional fixed designs, although additional considerations may be required for particular types of adaptations. For instance, drug supply for multi-arm studies is more complex as imbalances between centres can be larger and discontinuing arms will alter the drug demand in a difficult-to-predict manner. For trials that allow the ratio at which patients are allocated to each treatment to change once the trial is under way, it is especially important that there is a bespoke central system for randomisation. This will ensure that randomisation errors are minimised and that drug supply requirements can be communicated promptly to pharmacies dispensing study medication.

Various AD methods have been implemented in validated and easy-to-use statistical software packages over the past decade [21, 56, 57]. However, especially for novel ADs, off-the-shelf software may not be readily available, in which case quality control and validation of self-written programmes will take additional time and resources.

In this section, we have highlighted some of the considerations necessary when embarking on an AD. They are, of course, far from comprehensive and will depend on the type of adaptation(s) implemented. All these hurdles, however, have been overcome in many trials in practice. Table 1 lists just a few examples of successful AD trials. Practical challenges with ADs have also been discussed, e.g. in [46, 58–66], and practical experiences are described in [64, 67–69].

## Interpretation of trial results

In addition to these practical challenges around planning and running a trial, ADs also require some extra care when making sense of trial results. The formal numerical analysis of trial data will likely be undertaken by a statistician. We recommend consulting someone with expertise in and experience of ADs well enough in advance. The statistician can advise on appropriate analysis methods and assist with drafting the statistical analysis plan as well as pre-trial simulation studies to assess the statistical and operating characteristics of the proposed design, if needed.

While it may not be necessary for clinicians to comprehend advanced statistical techniques in detail, we believe that all investigators should be fully aware of the design’s implications and possible pitfalls in interpreting and reporting the findings correctly. In the following, we highlight how ADs may lead to issues with interpretability. We split them into statistical and non-statistical issues and consider how they may affect the interpretation of results as well as their subsequent reporting, e.g. in journal papers. Based on the discussion of these issues, in the next section we will identify limitations in how ADs are currently reported and make recommendations for improvement.

### Statistical issues

For a fixed randomised controlled trial (RCT) analysed using traditional statistics, it is common to present the estimated treatment effect (e.g. difference in proportions or means between treatment groups) alongside a 95% CI and *p* value. The latter is a summary measure of a hypothesis test whether the treatment effect is ‘significantly’ different from the null effect (e.g. the difference in means being zero) and is typically compared to a pre-specified ‘significance’ level (e.g. 5%). Statistical analyses of fixed RCTs will, in most cases, lead to treatment effect estimates, CIs and *p* values that have desirable and well-understood statistical properties:

1. Estimates will be *unbiased*, meaning that if the study were to be repeated many times according to the same protocol, the average estimate would be equal to the true treatment effect.
2. CIs will have *correct coverage*, meaning that if the study were to be repeated many times according to the same protocol, 95% of all 95% CIs calculated would contain the true treatment effect.
3. *p* values will be *well-calibrated*, meaning that when there is no effect of treatment, the chance of observing a *p* value less than 0.05 is exactly 5%.

These are by no means the only relevant criteria for assessing the performance of a trial design. Other metrics include the accuracy of estimation (e.g. mean squared error), the probability of identifying the true best treatment (especially with MAMS designs) and the ability to treat patients effectively within the trial (e.g. in dose-escalation studies). ADs usually perform considerably better than non-ADs in terms of these other criteria, which are also of more direct interest to patients. However, the three statistical properties listed above and also in Table 2 are essential requirements of regulators [40–42] and other stakeholders for accepting a (novel) design method.

**Table 2:** Important statistical quantities for reporting a clinical trial, and how they may be affected by an adaptive design

Statistical quantity	Fixed-design RCT property	Issue with adaptive design	Potential solution
Effect estimate	Unbiased: on average (across many trials) the effect estimate will have the same mean as the true value	Estimated treatment effect using naive methods can be biased, with an incorrect mean value	Use adjusted estimators that eliminate or reduce bias; use simulation to explore the extent of bias
Confidence interval	Correct coverage: 95% CIs will on average contain the true effect 95% of the time	CIs computed in the traditional way can have incorrect coverage	Use improved CIs that have correct or closer to correct coverage levels; use simulation to explore the actual coverage
*p* value	Well-calibrated: the nominal significance level used is equal to the type I error rate actually achieved	*p* values calculated in the traditional way may not be well-calibrated, i.e. could be conservative or anti-conservative	Use *p* values that have correct theoretical calibration; use simulation to explore the actual type I error rate of a design

*CI* confidence interval, *RCT* randomised controlled trial

The analysis of an AD trial often involves combining data from different stages, which can be done e.g. with the inverse normal method, *p* value combination tests or conditional error functions [70, 71]. It is still possible to compute the estimated treatment effect, its CI and a *p* value. If these quantities are, however, naively computed using the same methods as in a fixed-design trial, then they often lack the desirable properties mentioned above, depending on the nature of adaptations employed [72]. This is because the statistical distribution of the estimated treatment effect can be affected, sometimes strongly, by an AD [73]. The CI and *p* value usually depend on the treatment effect estimate and are, thus, also affected.

**Fig. 3:**
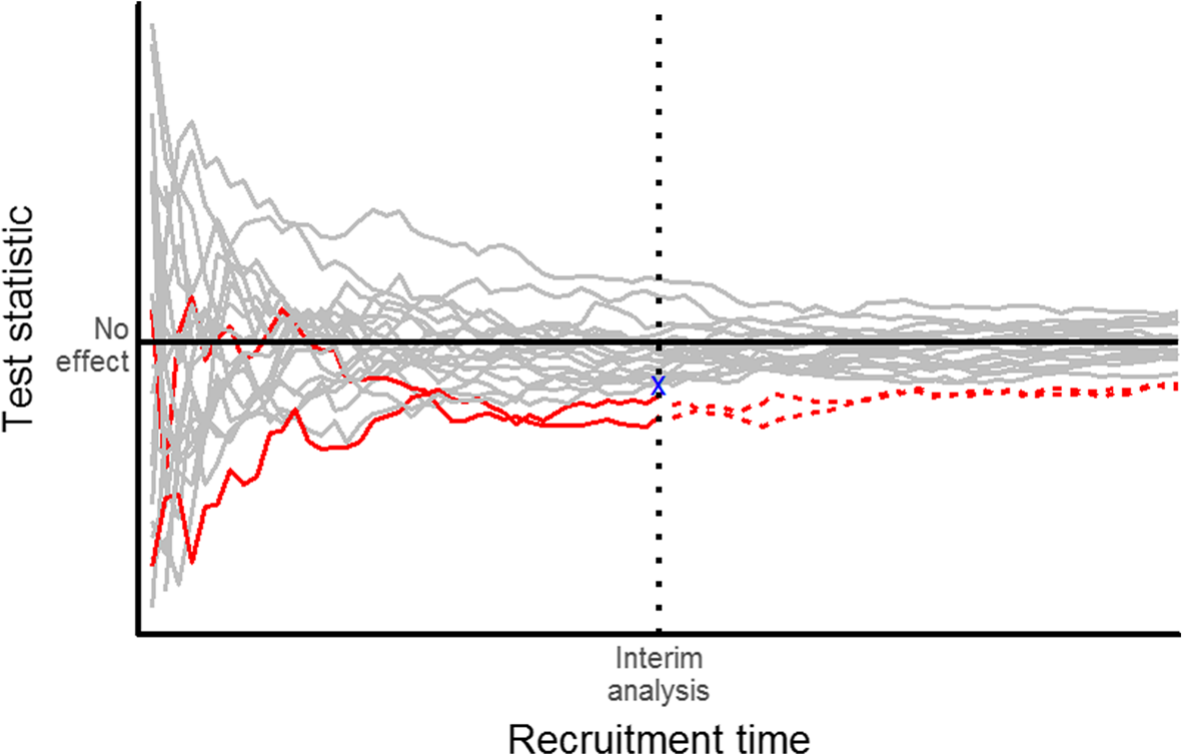
Illustration of bias introduced by early stopping for futility. This is for 20 simulated two-arm trials with no true treatment effect. The trajectories of the test statistics (as a standardised measure of the difference between treatments) are subject to random fluctuation. Two trials (red) are stopped early because their test statistics are below a pre-defined futility boundary (blue cross) at the interim analysis. Allowing trials with random highs at the interim to continue but terminating trials with random lows early will lead to an upward bias of the (average) treatment effect

As an example, consider a two-stage adaptive RCT that can stop early if the experimental treatment is doing poorly against the control at an interim analysis, based on a pre-specified stopping rule applied to data from patients assessed during the first stage. If the trial is not stopped early, the final estimated treatment effect calculated from all first- and second-stage patient data will be biased upwards. This is because the trial will stop early for futility at the first stage whenever the experimental treatment is—simply by chance—performing worse than average, and no additional second-stage data will be collected that could counterbalance this effect (via regression to the mean). The bottom line is that random lows are eliminated by the stopping rule but random highs are not, thus, biasing the treatment effect estimate upwards. See Fig. 3 for an illustration. This phenomenon occurs for a wide variety of ADs, especially when first-stage efficacy data are used to make adaptations such as discontinuing arms. Therefore, we provide several solutions that lead to sensible treatment effects estimates, CIs and *p* values from AD trials. See also Table 2 for an overview.

#### Treatment effect estimates

When stopping rules for an AD are clearly specified (as they should be), a variety of techniques are available to improve the estimation of treatment effects over naive estimators, especially for group-sequential designs. One approach is to derive an unbiased estimator [74–77]. Though unbiased, they will generally have a larger variance and thus, be less precise than other estimators. A second approach is to use an estimator that reduces the bias compared to the methods used for fixed-design trials, but does not necessarily completely eliminate it. Examples of this are the bias-corrected maximum likelihood estimator [78] and the median unbiased estimator [79]. Another alternative is to use shrinkage approaches for trials with multiple treatment arms [36, 80, 81]. In general, such estimators substantially reduce the bias compared to the naive estimator. Although they are not usually statistically unbiased, they have lower variance than the unbiased estimators [74, 82]. In trials with time-to-event outcomes, a follow-up to the planned end of the trial can markedly reduce the bias in treatment arms discontinued at interim [83].

An improved estimator of the treatment effect is not yet available for all ADs. In such cases, one may empirically adjust the treatment effect estimator via bootstrapping [84], i.e. by repeatedly sampling from the data and calculating the estimate for each sample, thereby building up a ‘true’ distribution of the estimator that can be used to adjust it. Simulations can then be used to assess the properties of this bootstrap estimator. The disadvantage of bootstrapping is that it may require a lot of computing power, especially for more complex ADs.

#### Confidence intervals

For some ADs, there are CIs that have the correct coverage level taking into account the design used [18, 19, 85, 86], including simple repeated CIs [87]. If a particular AD does not have a method that can be readily applied, then it is advisable to carry out simulations at the design stage to see whether the coverage of the naively found CIs deviates considerably from the planned level. In that case, a bootstrap procedure could be applied for a wide range of designs if this is not too computationally demanding.

#### *p* values

A *p* value is often presented alongside the treatment effect estimate and CI as it helps to summarise the level of evidence against the null hypothesis. For certain ADs, such as group-sequential methods, one can order the possible trial outcomes by how ‘extreme’ they are in terms of the strength of evidence they represent against the null hypothesis. In a fixed-design trial, this is simply the magnitude of the test statistic. However, in an AD that allows early stopping for futility or efficacy, it is necessary to distinguish between different ways in which the null hypothesis might be rejected [73]. For example, we might conclude that if a trial stops early and rejects the null hypothesis, this is more ‘extreme’ evidence against the null than if the trial continues to the end and only then rejects it. There are several different ways that data from an AD may be ordered, and the *p* value found (and also the CI) may depend on which method is used. Thus, it is essential to pre-specify which method will be used and to provide some consideration of the sensitivity of the results to the method.

#### Type I error rates

The total probability of rejecting the null hypothesis (type I error rate) is an important quantity in clinical trials, especially for phase III trials where a type I error may mean an ineffective or harmful treatment will be used in practice. In some ADs, a single null hypothesis is tested but the actual type I error rate is different from the planned level specified before the trial, unless a correction is performed. As an example, if unblinded data (with knowledge or use of treatment allocation such that the interim treatment effect can be inferred) are used to adjust the sample size at the interim, then the inflation to the planned type I error can be substantial and needs to be accounted for [16, 34, 35, 88]. On the other hand, blinded sample size re-estimation (done without knowledge or use of treatment allocation) usually has a negligible impact on the type I error rate and inference when performed with a relatively large sample size, but inflation can still occur [89, 90].

#### Multiple hypothesis testing

In some ADs, multiple hypotheses are tested (e.g. in MAMS trials), or the same hypothesis is re-tested multiple times (e.g. interim and final analyses [91]), or the effects on the primary and key secondary endpoints may be tested group-sequentially [92, 93], all of which may lead to type I error rate inflation. In any (AD or non-AD) trial, the more (often the) null hypotheses are tested, the higher the chance that one will be incorrectly rejected. To control the overall (family-wise) type I error rate at a fixed level (say, 5%), adjustment for multiple testing is necessary [94]. This can sometimes be done with relatively simple methods [95]; however, it may not be possible for all multiple testing procedures to derive corresponding useful CIs.

In a MAMS setting, adjustment is viewed as being particularly important when the trial is confirmatory and when the research arms are different doses or regimens of the same treatment, whereas in some other cases, it might not be considered essential, e.g. when the research treatments are substantially different, particularly if developed by different groups [96]. When making a decision about whether to adjust for multiplicity, it may help to think what adjustment would have been required had the results of the equivalent trials been conducted as separate two-arm trials. Regulatory guidance is commonly interpreted as encouraging strict adjustment for multiple testing within a single trial [97–99].

#### Bayesian methods

While this paper focuses on frequentist (classical) statistical methods for trial design and analysis, there is also a wealth of Bayesian AD methods [100] that are increasingly being applied in clinical research [23]. Bayesian designs are much more common for early-phase dose escalation [101, 102] and adaptive randomisation [103] but are gaining popularity also in confirmatory settings [104], such as seamless phase II/III trials [105] and in umbrella or basket trials [106]. Bayesian statistics and adaptivity go very well together [4]. For instance, taking multiple looks at the data is (statistically) unproblematic as it does not have to be adjusted for separately in a Bayesian framework.

Although Bayesian statistics is by nature not concerned with type I error rate control or *p* values, it is common to evaluate and report the frequentist operating characteristics of Bayesian designs, such as power and type I error rate [107–109]. Consider e.g. the frequentist and Bayesian interpretations of group-sequential designs [110–112]. Moreover, there are some hybrid AD methods that blend frequentist and Bayesian aspects [113–115].

### Non-statistical issues

Besides these statistical issues, the interpretability of results may also be affected by the way triallists conduct an AD trial, in particular with respect to mid-trial data analyses. Using interim data to modify study aspects may raise anxiety in some research stakeholders due to the potential introduction of operational bias. Knowledge, leakage or mere speculation of interim results could alter the behaviour of those involved in the trial, including investigators, patients and the scientific community [116, 117]. Hence, it is vital to describe the processes and procedures put in place to minimise potential operational bias. Triallists, as well as consumers of trial reports, should give consideration to:

- who had access to interim data or performed interim analyses
- how the results were shared and confidentiality maintained
- what the role of the sponsor was in the decision-making process.

The importance of confidentiality and models for monitoring AD trials have been discussed [46, 118].

Inconsistencies in the conduct of the trial across different stages (e.g. changes to care given and how outcomes are assessed) may also introduce operational bias, thus, undermining the internal and external validity and therefore, the credibility of trial findings. As an example, modifications of eligibility criteria might lead to a shift in the patient population over time, and results may depend on whether patients were recruited before or after the interim analysis. Consequently, the ability to combine results across independent interim stages to assess the overall treatment effect becomes questionable. Heterogeneity between the stages of an AD trial could also arise when the trial begins recruiting from a limited number of sites (in a limited number of countries), which may not be representative of all the sites that will be used once recruitment is up and running [55].

Difficulties faced in interpreting research findings with heterogeneity across interim stages have been discussed in detail [119–123]. Although it is hard to distinguish heterogeneity due to change from that influenced by operational bias, we believe there is a need to explore stage-wise heterogeneity by presenting key patient characteristics and results by independent stages and treatment groups.

## Reporting adaptive designs

High-quality reporting of results is a vital part of running any successful trial [124]. The reported findings need to be credible, transparent and repeatable. Where there are potential biases, the report should highlight them, and it should also comment on how sensitive the results are to the assumptions made in the statistical analysis. Much effort has been made to improve the reporting quality of traditional clinical trials. One high-impact initiative is the CONSORT (Consolidated Standards of Reporting Trials) statement [125], which itemises a minimum set of information that should be included in reports of RCTs.

We believe that to report an AD trial in a credible, transparent and repeatable fashion, additional criteria beyond those in the core CONSORT statement are required. Recent work has discussed the reporting of AD trials with examples of and recommendations for minimum standards [126–128] and identified several items in the CONSORT check list as relevant when reporting an AD trial [129, 130].

Mindful of the statistical and operational pitfalls discussed in the previous section, we have compiled a list of 11 reporting items that we consider essential for AD trials, along with some explanations and examples. Given the limited word counts of most medical journals, we acknowledge that a full description of all these items may need to be included as supplementary material. However, sufficient information must be provided in the main body, with references to additional material.

### Rationale for the AD, research objectives and hypotheses

Especially for novel and ‘less well-understood’ ADs (a term coined in [41]), a clear rationale for choosing an AD instead of a more traditional design approach should be given, explaining the potential added benefits of the adaptation(s). This will enable readers and reviewers to gauge the appropriateness of the design and interpret its findings correctly. Research objectives and hypotheses should be set out in detail, along with how the chosen AD suits them. Reasons for using more established ADs have been discussed in the literature, e.g. why to prefer the continual reassessment method (CRM) over a traditional 3+3 design for dose escalation [131, 132], or why to use seamless and MAMS designs [133, 134]. The choice of routinely used ADs, such as CRM for dose escalation or group-sequential designs, should be self-evident and need not be justified every time.

### Type and scope of AD

A trial report should not only state the type of AD used but also describe its scope adequately. This allows the appropriateness of the statistical methods used to be assessed and the trial to be replicated. The scope relates to what the adaptation(s) encompass, such as terminating futile treatment arms or selecting the best performing treatment in a MAMS design. The scope of ADs with varying objectives is broad and can sometimes include multiple adaptations aimed at addressing multiple objectives in a single trial.

### Sample sizes

In addition to reporting the overall planned and actually recruited sample sizes as in any RCT, AD trial reports should provide information on the timing of interim analyses (e.g. in terms of fractions of total number of patients, or number of events for survival data) and how many patients contributed to each interim analysis.

### Adaptation criteria

Transparency with respect to adaptation procedures is crucial [135]. Hence, reports should include the decision rules used, their justification and timing as well as the frequency of interim analyses. It is important for the research team, including the clinical and statistical researchers, to discuss adaptation criteria at the planning stage and to consider the validity and clinical interpretation of the results.

### Simulations and pre-trial work

For ‘well-understood’ ADs, such as standard group-sequential methods, referencing peer-reviewed publications and the statistical software used will be sufficient to justify the validity of the design. Some ADs, however, may require simulation work under a number of scenarios to:

- evaluate the statistical properties of the design such as (family-wise) type I error rate, sample size and power
- assess the potential bias that may result from the statistical estimation procedure
- explore the impact of (not) implementing adaptations on both statistical properties and operational characteristics.

It is important to provide clear simulation objectives, a rationale for the scenarios investigated and evidence showing that the desired statistical properties have been preserved. The simulation protocol and report, as well as any software code used to generate the results, should be made accessible.

### Statistical methods

As ADs may warrant special methods to produce valid inference (see Table 2), it is particularly important to state how treatment effect estimates, CIs and *p* values were obtained. In addition, traditional naive estimates could be reported alongside adjusted estimates. Whenever data from different stages are combined in the analysis, it is important to disclose the combination method used as well as the rationale behind it.

### Heterogeneity

Heterogeneity of the baseline characteristics of study participants or of the results across interim stages and/or study sites may undermine the interpretation and credibility of results for some ADs. Reporting the following, if appropriate for the design used, could provide some form of assurance to the scientific research community:

- important baseline summaries of participants recruited in different stages
- summaries of site contributions to interim results
- exploration of heterogeneity of results across stages or sites
- path of interim results across stages, even if only using naive treatment effects and CIs.

Nonetheless, differentiating between randomly occurring and design-induced heterogeneity or population drift is tough, and even standard fixed designs are not immune to this problem.

### Unplanned modifications

Prospective planning of an AD is important for credibility and regulatory considerations [41]. However, as in any other (non-AD) trial, some events not envisaged during the course of the trial may call for changes to the design that are outside the scope of a priori planned adaptations, or there may be a failure to implement planned adaptations. Questions may be raised regarding the implications of such unplanned ad hoc modifications. Is the planned statistical framework still valid? Were the changes driven by potential bias? Are the results still interpretable in relation to the original research question? Thus, any unplanned modifications must be stated clearly, with an explanation as to why they were implemented and how they may impact the interpretation of trial results.

### Interpretability of results

As highlighted earlier, adaptations should be motivated by the need to address specific research objectives. In the context of the trial conducted and its observed results, triallists should discuss the interpretability of results in relation to the original research question(s). In particular, who the study results apply to should be considered. For instance, subgroup selection, enrichment and biomarker ADs are motivated by the need to characterise patients who are most likely to benefit from investigative treatments. Thus, the final results may apply only to patients with specific characteristics and not to the general or enrolled population.

### Lessons learned

What worked well? What went wrong? What could have been done differently? We encourage the discussion of all positive, negative and perhaps surprising lessons learned over the course of an AD trial. Sharing practical experiences with AD methods will help inform the design, planning and conduct of future trials and is, thus, a key element in ensuring researchers are competent and confident enough to apply ADs in their own trials [27]. For novel cutting-edge designs especially, we recommend writing up and publishing these experiences as a statistician-led stand-alone paper.

### Indexing

Terms such as ‘adaptive design’, ‘adaptive trial design’ or ‘adaptive trial’ should appear in the title and/or abstract or at least among the keywords of the trial report and key publications. Otherwise, retrieving and identifying AD trials in the literature and clinical trial registers will be a major challenge for researchers and systematic reviewers [28].

## Discussion

We wrote this paper to encourage the wider use of ADs with pre-planned opportunities to make design changes in clinical trials. Although there are a few practical stumbling blocks on the way to a good AD trial, they can almost always be overcome with careful planning. We have highlighted some pivotal issues around funding, communication and implementation that occur in many AD trials. When in doubt about a particular design aspect, we recommend looking up and learning from examples of trials that have used similar designs. As AD methods are beginning to find their way into clinical research, more case studies will become available for a wider range of applications. Practitioners clearly need to publish more of their examples. Table 1 lists a very small selection.

Over the last two decades, we have seen and been involved with dozens of trials where ADs have sped up, shortened or otherwise improved trials. Thus, our key message is that ADs should no longer be ‘a dream for statisticians only’ [23] but rather a part of every clinical investigator’s methodological tool belt. That is, however, not to say that all trials should be adaptive. Under some circumstances, an AD would be nonsensical, e.g. if the outcome measure of interest takes so long to record that there is basically no time for the adaptive changes to come into effect before the trial ends. Moreover, it is important to realise that pre-planned adaptations are a safeguard against shaky assumptions at the planning stage, not a means to rescue an otherwise poorly designed trial.

ADs indeed carry a risk of introducing bias into a trial. That being said, avoiding ADs for fear of biased results is uncalled for. The magnitude of the statistical bias is practically negligible in many cases, and there are methods to counteract it. The best way to minimise operational bias (which is by no means unique to ADs) is by rigorous planning and transparency. Measures such as establishing well-trained and well-informed IDMCs and keeping triallists blind to changes wherever possible, as well as clear and comprehensive reporting, will help build trust in the findings of an AD trial.

The importance of accurately reporting all design specifics, as well as the adaptations made and the trial results, cannot be overemphasised, especially since clear and comprehensive reports facilitate the learning for future (AD or non-AD) trials. Working through our list of recommendations should be a good starting point. These reporting items are currently being formalised, with additional input from a wide range of stakeholders, as an AD extension to the CONSORT reporting guidance and check list.

## Abbreviations

AD: Adaptive design
CARISA: Combination Assessment of Ranolazine In Stable Angina
CI: Confidence interval
CONSORT: Consolidated Standards of Reporting Trials
CRM: Continual reassessment method
IA: Idarubicin + ara-C
IDMC: Independent data monitoring committee
MAMS: Multi-arm multi-stage
RAR: Response-adaptive randomisation
RCT: Randomised controlled trial
TA: Troxacitabine + ara-C
TAILoR: Telmisartan and Insulin Resistance in HIV
TI: Troxacitabine + idarubicin
TSC: Trial steering committee

## References

[1] Friedman FL, Furberg CD, DeMets DL (2010). Fundamentals of clinical trials.

[2] Shih WJ (2006). Plan to be flexible: a commentary on adaptive designs. Biometrical J.

[3] Berry Consultants. What is adaptive design? 2016. http://www.berryconsultants.com/adaptive-designs. Accessed 7 Jul 2017.

[4] Campbell G (2013). Similarities and differences of Bayesian designs and adaptive designs for medical devices: a regulatory view. Stat Biopharm Res.

[5] Chow SC, Chang M (2012). Adaptive design methods in clinical trials.

[6] Morgan CC (2003). Sample size re-estimation in group-sequential response-adaptive clinical trials. Stat Med.

[7] Parmar MKB, Barthel FMS, Sydes M, Langley R, Kaplan R, Eisenhauer E (2008). Speeding up the evaluation of new agents in cancer. J Natl Cancer Inst.

[8] Zohar S, Chevret S (2007). Recent developments in adaptive designs for phase I/II dose-finding studies. J Biopharm Stat.

[9] Sverdlov O, Wong WK (2014). Novel statistical designs for phase I/II and phase II clinical trials with dose-finding objectives. Ther Innov Regul Sci.

[10] Maca J, Bhattacharya S, Dragalin V, Gallo P, Krams M (2006). Adaptive seamless phase II/III designs—background, operational aspects, and examples. Drug Inf J.

[11] Stallard N, Todd S (2011). Seamless phase II/III designs. Stat Methods Med Res.

[12] Chow SC, Chang M, Pong A (2005). Statistical consideration of adaptive methods in clinical development. J Biopharm Stat.

[13] Fleming TR, Sharples K, McCall J, Moore A, Rodgers A, Stewart R (2008). Maintaining confidentiality of interim data to enhance trial integrity and credibility. Clin Trials.

[14] Bauer P, Koenig F, Brannath W, Posch M (2010). Selection and bias—two hostile brothers. Stat Med.

[15] Posch M, Maurer W, Bretz F (2011). Type I error rate control in adaptive designs for confirmatory clinical trials with treatment selection at interim. Pharm Stat.

[16] Graf AC, Bauer P (2011). Maximum inflation of the type 1 error rate when sample size and allocation rate are adapted in a pre-planned interim look. Stat Med.

[17] Graf AC, Bauer P, Glimm E, Koenig F (2014). Maximum type 1 error rate inflation in multiarmed clinical trials with adaptive interim sample size modifications. Biometrical J.

[18] Magirr D, Jaki T, Posch M, Klinglmueller F (2013). Simultaneous confidence intervals that are compatible with closed testing in adaptive designs. Biometrika.

[19] Kimani PK, Todd S, Stallard N (2014). A comparison of methods for constructing confidence intervals after phase II/III clinical trials. Biometrical J.

[20] Lorch U, Berelowitz K, Ozen C, Naseem A, Akuffo E, Taubel J (2012). The practical application of adaptive study design in early phase clinical trials: a retrospective analysis of time savings. Eur J Clin Pharmacol.

[21] Bauer P, Bretz F, Dragalin V, König F, Wassmer G (2016). Twenty-five years of confirmatory adaptive designs: opportunities and pitfalls. Stat Med.

[22] Lee JJ, Siu LL, Le Tourneau C (2009). Dose escalation methods in phase I cancer clinical trials. J Natl Cancer Inst.

[23] Chevret S (2012). Bayesian adaptive clinical trials: a dream for statisticians only?. Stat Med.

[24] Jaki T (2013). Uptake of novel statistical methods for early-phase clinical studies in the UK public sector. Clin Trials.

[25] Morgan CC, Huyck S, Jenkins M, Chen L, Bedding A, Coffey CS (2014). Adaptive design: results of 2012 survey on perception and use. Ther Innov Regul Sci.

[26] Dimairo M, Boote J, Julious SA, Nicholl JP, Todd S (2015). Missing steps in a staircase: a qualitative study of the perspectives of key stakeholders on the use of adaptive designs in confirmatory trials. Trials.

[27] Dimairo M, Julious SA, Todd S, Nicholl JP, Boote J (2015). Cross-sector surveys assessing perceptions of key stakeholders towards barriers, concerns and facilitators to the appropriate use of adaptive designs in confirmatory trials. Trials.

[28] Hatfield I, Allison A, Flight L, Julious SA, Dimairo M (2016). Adaptive designs undertaken in clinical research: a review of registered clinical trials. Trials.

[29] Meurer WJ, Legocki L, Mawocha S, Frederiksen SM, Guetterman TC, Barsan W (2016). Attitudes and opinions regarding confirmatory adaptive clinical trials: a mixed methods analysis from the Adaptive Designs Accelerating Promising Trials into Treatments (ADAPT-IT) project. Trials.

[30] Chaitman BR, Pepine CJ, Parker JO, Skopal J, Chumakova G, Kuch J (2004). Effects of ranolazine with atenolol, amlodipine, or diltiazem on exercise tolerance and angina frequency in patients with severe chronic angina: a randomized controlled trial. J Am Med Assoc.

[31] Pushpakom SP, Taylor C, Kolamunnage-Dona R, Spowart C, García-Fiñana M, Vora J (2015). Telmisartan and insulin resistance in HIV (TAILoR): protocol for a dose-ranging phase II randomised open-labelled trial of telmisartan as a strategy for the reduction of insulin resistance in HIV-positive individuals on combination antiretroviral therapy. BMJ Open.

[32] Magirr D, Jaki T, Whitehead J (2012). A generalized Dunnett test for multi-arm multi-stage clinical studies with treatment selection. Biometrika.

[33] Giles FJ, Kantarjian HM, Cortes JE, Garcia-Manero G, Verstovsek S, Faderl S (2003). Adaptive randomized study of idarubicin and cytarabine versus troxacitabine and cytarabine versus troxacitabine and idarubicin in untreated patients 50 years or older with adverse karyotype acute myeloid leukemia. J Clin Oncol.

[34] Mehta CR, Pocock SJ (2011). Adaptive increase in sample size when interim results are promising: a practical guide with examples. Stat Med.

[35] Jennison C, Turnbull BW (2015). Adaptive sample size modification in clinical trials: start small then ask for more?. Stat Med.

[36] Bowden J, Brannath W, Glimm E (2014). Empirical Bayes estimation of the selected treatment mean for two-stage drop-the-loser trials: a meta-analytic approach. Stat Med.

[37] Mason AJ, Gonzalez-Maffe J, Quinn K, Doyle N, Legg K, Norsworthy P (2017). Developing a Bayesian adaptive design for a phase I clinical trial: a case study for a novel HIV treatment. Stat Med.

[38] Wellcome Trust. Joint Global Health Trials scheme. 2017. https://wellcome.ac.uk/funding/joint-global-health-trials-scheme. Accessed 7 Jul 2017.

[39] National Institutes of Health. NIH Planning Grant Program (R34). 2014. https://grants.nih.gov/grants/funding/r34.htm. Accessed 7 Jul 2017.

[40] European Medicines Agency. Reflection paper on methodological issues in confirmatory clinical trials planned with an adaptive design. 2007. http://www.ema.europa.eu/docs/en_GB/document_library/Scientific_guideline/2009/09/WC500003616.pdf. Accessed 7 Jul 2017.

[41] US Food & Drug Administration. Adaptive design clinical trials for drugs and biologics: guidance for industry (draft). 2010. https://www.fda.gov/downloads/drugs/guidances/ucm201790.pdf. Accessed 7 Jul 2017.

[42] Food US & Drug Administration. Adaptive designs for medical device clinical studies: guidance for industry and Food and Drug Administration staff.2016. https://www.fda.gov/downloads/medicaldevices/deviceregulationandguidance/guidancedocuments/ucm446729.pdf. Accessed 7 Jul 2017.

[43] Gaydos B, Koch A, Miller F, Posch M, Vandemeulebroecke M, Wang SJ (2012). Perspective on adaptive designs: 4 years European Medicines Agency reflection paper, 1 year draft US FDA guidance—where are we now?. Clin Investig.

[44] Elsäßer A, Regnstrom J, Vetter T, Koenig F, Hemmings RJ, Greco M (2014). Adaptive clinical trial designs for European marketing authorization: a survey of scientific advice letters from the European Medicines Agency. Trials.

[45] DeMets DL, Fleming TR (2004). The independent statistician for data monitoring committees. Stat Med.

[46] Gallo P (2006). Operational challenges in adaptive design implementation. Pharm Stat.

[47] Grant AM, Altman DG, Babiker AG, Campbell MK, Clemens F, Darbyshire JH (2005). A proposed charter for clinical trial data monitoring committees: helping them to do their job well. Lancet.

[48] Antonijevic Z, Gallo P, Chuang-Stein C, Dragalin V, Loewy J, Menon S (2013). Views on emerging issues pertaining to data monitoring committees for adaptive trials. Ther Innov Regul Sci.

[49] Sanchez-Kam M, Gallo P, Loewy J, Menon S, Antonijevic Z, Christensen J (2014). A practical guide to data monitoring committees in adaptive trials. Ther Innov Regul Sci.

[50] DeMets DL, Ellenberg SS (2016). Data monitoring committees—expect the unexpected. N Engl J Med.

[51] Calis KA, Archdeacon P, Bain R, DeMets D, Donohue M, Elzarrad MK (2017). Recommendations for data monitoring committees from the Clinical Trials Transformation Initiative. Clin Trials.

[52] Conroy EJ, Harman NL, Lane JA, Lewis SC, Murray G, Norrie J (2015). Trial steering committees in randomised controlled trials: a survey of registered clinical trials units to establish current practice and experiences. Clin Trials.

[53] Harman NL, Conroy EJ, Lewis SC, Murray G, Norrie J, Sydes MR (2015). Exploring the role and function of trial steering committees: results of an expert panel meeting. Trials.

[54] Daykin A, Selman LE, Cramer H, McCann S, Shorter GW, Sydes MR (2016). What are the roles and valued attributes of a trial steering committee? Ethnographic study of eight clinical trials facing challenges. Trials.

[55] He W, Gallo P, Miller E, Jemiai Y, Maca J, Koury K (2017). Addressing challenges and opportunities of ‘less well-understood’ adaptive designs. Ther Innov Regul Sci.

[56] Zhu L, Ni L, Yao B (2011). Group sequential methods and software applications. Am Stat.

[57] Tymofyeyev Y, He W, Pinheiro J, Kuznetsova OM (2014). A review of available software and capabilities for adaptive designs. Practical considerations for adaptive trial design and implementation.

[58] Gallo P, Chuang-Stein C, Dragalin V, Gaydos B, Krams M, Pinheiro J (2006). Adaptive designs in clinical drug development—an executive summary of the PhRMA Working Group. J Biopharm Stat.

[59] Quinlan J, Krams M (2006). Implementing adaptive designs: logistical and operational considerations. Drug Inf J.

[60] Chow SC, Chang M (2008). Adaptive design methods in clinical trials—a review. Orphanet J Rare Dis.

[61] Bretz F, Koenig F, Brannath W, Glimm E, Posch M (2009). Adaptive designs for confirmatory clinical trials. Stat Med.

[62] Quinlan J, Gaydos B, Maca J, Krams M (2010). Barriers and opportunities for implementation of adaptive designs in pharmaceutical product development. Clin Trials.

[63] He W, Kuznetsova OM, Harmer M, Leahy C, Anderson K, Dossin N (2012). Practical considerations and strategies for executing adaptive clinical trials. Ther Innov Regul Sci.

[64] He W, Pinheiro J, Kuznetsova OM. Practical considerations for adaptive trial design and implementation. New York: Springer; 2014.

[65] Curtin F, Heritier S (2017). The role of adaptive trial designs in drug development. Expert Rev Clin Pharmacol.

[66] Petroni GR, Wages NA, Paux G, Dubois F (2017). Implementation of adaptive methods in early-phase clinical trials. Stat Med.

[67] Sydes MR, Parmar MKB, James ND, Clarke NW, Dearnaley DP, Mason MD (2009). Issues in applying multi-arm multi-stage methodology to a clinical trial in prostate cancer: the MRC STAMPEDE trial. Trials.

[68] Spencer K, Colvin K, Braunecker B, Brackman M, Ripley J, Hines P (2012). Operational challenges and solutions with implementation of an adaptive seamless phase 2/3 study. J Diabetes Sci Technol.

[69] Miller E, Gallo P, He W, Kammerman LA, Koury K, Maca J (2017). DIA’s Adaptive Design Scientific Working Group (ADSWG): best practices case studies for ‘less well-understood’ adaptive designs. Ther Innov Regul Sci.

[70] Schäfer H, Timmesfeld N, Müller HH (2006). An overview of statistical approaches for adaptive designs and design modifications. Biom J.

[71] Wassmer G, Brannath W (2016). Group sequential and confirmatory adaptive designs in clinical trials.

[72] Ellenberg SS, DeMets DL, Fleming TR (2010). Bias and trials stopped early for benefit. J Am Med Assoc.

[73] Jennison C, Turnbull BW (2000). Analysis following a sequential test. Group sequential methods with applications to clinical trials.

[74] Emerson SS, Fleming TR (1990). Parameter estimation following group sequential hypothesis testing. Biometrika.

[75] Liu A, Hall WJ (1999). Unbiased estimation following a group sequential test. Biometrika.

[76] Bowden J, Glimm E (2008). Unbiased estimation of selected treatment means in two-stage trials. Biometrical J.

[77] Bowden J, Glimm E (2014). Conditionally unbiased and near unbiased estimation of the selected treatment mean for multistage drop-the-losers trials. Biometrical J.

[78] Whitehead J (1986). On the bias of maximum likelihood estimation following a sequential test. Biometrika.

[79] Jovic G, Whitehead J (2010). An exact method for analysis following a two-stage phase II cancer clinical trial. Stat Med.

[80] Carreras M, Brannath W (2013). Shrinkage estimation in two-stage adaptive designs with midtrial treatment selection. Stat Med.

[81] Brueckner M, Titman A, Jaki T (2017). Estimation in multi-arm two-stage trials with treatment selection and time-to-event endpoint. Stat Med.

[82] Bowden J, Wason J (2012). Identifying combined design and analysis procedures in two-stage trials with a binary end point. Stat Med.

[83] Choodari-Oskooei B, Parmar MK, Royston P, Bowden J (2013). Impact of lack-of-benefit stopping rules on treatment effect estimates of two-arm multi-stage (TAMS) trials with time to event outcome. Trials.

[84] Efron B, Tibshirani RJ (1993). An introduction to the bootstrap.

[85] Gao P, Liu L, Mehta C (2013). Exact inference for adaptive group sequential designs. Stat Med.

[86] Kimani PK, Todd S, Stallard N (2015). Estimation after subpopulation selection in adaptive seamless trials. Stat Med.

[87] Jennison C, Turnbull BW (1989). Interim analyses: the repeated confidence interval approach. J R Stat Soc Series B Stat Methodol.

[88] Proschan MA, Hunsberger SA (1995). Designed extension of studies based on conditional power. Biometrics.

[89] Kieser M, Friede T (2003). Simple procedures for blinded sample size adjustment that do not affect the type I error rate. Stat Med.

[90] żebrowska M, Posch M, Magirr D (2016). Maximum type I error rate inflation from sample size reassessment when investigators are blind to treatment labels. Stat Med.

[91] Bratton DJ, Parmar MKB, Phillips PPJ, Choodari-Oskooei B (2016). Type I error rates of multi-arm multi-stage clinical trials: strong control and impact of intermediate outcomes. Trials.

[92] Glimm E, Maurer W, Bretz F (2010). Hierarchical testing of multiple endpoints in group-sequential trials. Stat Med.

[93] Ye Y, Li A, Liu L, Yao B (2013). A group sequential Holm procedure with multiple primary endpoints. Stat Med.

[94] Maurer W, Branson M, Posch M, Dmitrienko A, Tamhane AC, Bretz F (2010). Adaptive designs and confirmatory hypothesis testing. Multiple testing problems in pharmaceutical statistics.

[95] Posch M, Koenig F, Branson M, Brannath W, Dunger-Baldauf C, Bauer P (2005). Testing and estimation in flexible group sequential designs with adaptive treatment selection. Stat Med.

[96] Wason JMS, Stecher L, Mander AP (2014). Correcting for multiple-testing in multi-arm trials: is it necessary and is it done?. Trials.

[97] Wang SJ, Hung HMJ, O’Neill R (2011). Regulatory perspectives on multiplicity in adaptive design clinical trials throughout a drug development program. J Biopharm Stat.

[98] European Medicines Agency. Guideline on multiplicity issues in clinical trials (draft). 2017. http://www.ema.europa.eu/docs/en_GB/document_library/Scientific_guideline/2017/03/WC500224998.pdf. Accessed 7 Jul 2017.

[99] US Food & Drug Administration. Multiple endpoints in clinical trials: guidance for industry (draft). 2017. https://www.fda.gov/downloads/Drugs/GuidanceComplianceRegulatoryInformation/Guidances/UCM536750.pdf. Accessed 7 Jul 2017.

[100] Berry SM, Carlin BP, Lee JJ, Müller P (2010). Bayesian adaptive methods for clinical trials.

[101] Chevret S (2006). Statistical methods for dose-finding experiments.

[102] Cheung YK (2011). Dose finding by the continual reassessment method.

[103] Thall PF, Wathen JK (2007). Practical Bayesian adaptive randomisation in clinical trials. Eur J Cancer.

[104] Jansen JO, Pallmann P, MacLennan G, Campbell MK (2017). Bayesian clinical trial designs: another option for trauma trials?. J Trauma Acute Care Surg.

[105] Kimani PK, Glimm E, Maurer W, Hutton JL, Stallard N (2012). Practical guidelines for adaptive seamless phase II/III clinical trials that use Bayesian methods. Stat Med.

[106] Liu S, Lee JJ (2015). An overview of the design and conduct of the BATTLE trials. Chin Clin Oncol.

[107] Cheng Y, Shen Y (2005). Bayesian adaptive designs for clinical trials. Biometrika.

[108] Lewis RJ, Lipsky AM, Berry DA (2007). Bayesian decision-theoretic group sequential clinical trial design based on a quadratic loss function: a frequentist evaluation. Clin Trials.

[109] Ventz S, Trippa L (2015). Bayesian designs and the control of frequentist characteristics: a practical solution. Biometrics.

[110] Emerson SS, Kittelson JM, Gillen DL (2007). Frequentist evaluation of group sequential clinical trial designs. Stat Med.

[111] Emerson SS, Kittelson JM, Gillen DL (2007). Bayesian evaluation of group sequential clinical trial designs. Stat Med.

[112] Gsponer T, Gerber F, Bornkamp B, Ohlssen D, Vandemeulebroecke M, Schmidli H (2014). A practical guide to Bayesian group sequential designs. Pharm Stat.

[113] Stallard N, Whitehead J, Cleall S (2005). Decision-making in a phase II clinical trial: a new approach combining Bayesian and frequentist concepts. Pharm Stat.

[114] Dong G, Shih WJ, Moore D, Quan H, Marcella S (2012). A Bayesian-frequentist two-stage single-arm phase II clinical trial design. Stat Med.

[115] Hartley AM (2012). Adaptive blinded sample size adjustment for comparing two normal means—a mostly Bayesian approach. Pharm Stat.

[116] Gallo P (2006). Confidentiality and trial integrity issues for adaptive designs. Drug Inf J.

[117] Broglio KR, Stivers DN, Berry DA (2014). Predicting clinical trial results based on announcements of interim analyses. Trials.

[118] Chow SC, Corey R, Lin M (2012). On the independence of data monitoring committee in adaptive design clinical trials. J Biopharm Stat.

[119] Friede T, Henderson R (2009). Exploring changes in treatment effects across design stages in adaptive trials. Pharm Stat.

[120] Gallo P, Chuang-Stein C (2009). What should be the role of homogeneity testing in adaptive trials?. Pharm Stat.

[121] Gonnermann A, Framke T, Großhennig A, Koch A (2015). No solution yet for combining two independent studies in the presence of heterogeneity. Stat Med.

[122] Parker RA (2010). Testing for qualitative interactions between stages in an adaptive study. Stat Med.

[123] Wang SJ, Brannath W, Brückner M, Hung HMJ, Koch A (2013). Unblinded adaptive statistical information design based on clinical endpoint or biomarker. Stat Biopharm Res.

[124] Jüni P, Altman DG, Egger M (2001). Systematic reviews in health care: assessing the quality of controlled clinical trials. BMJ.

[125] Schulz KF, Altman DG, Moher D (2010). CONSORT 2010 statement: updated guidelines for reporting parallel group randomised trials. BMJ.

[126] Bauer P, Einfalt J (2006). Application of adaptive designs—a review. Biometrical J.

[127] Detry MA, Lewis RJ, Broglio KR, Connor JT, Berry SM, Berry DA. Standards for the design, conduct, and evaluation of adaptive randomized clinical trials. Washington: Patient-Centered Outcomes Research Institute; 2012. http://www.pcori.org/assets/Standards-for-the-Design-Conduct-and-Evaluation-of-Adaptive-Randomized-Clinical-Trials.pdf. Accessed 7 Jul 2017.

[128] Lorch U, O’Kane M, Taubel J (2014). Three steps to writing adaptive study protocols in the early phase clinical development of new medicines. BMC Med Res Methodol.

[129] Stevely A, Dimairo M, Todd S, Julious SA, Nicholl J, Hind D (2015). An investigation of the shortcomings of the CONSORT 2010 statement for the reporting of group sequential randomised controlled trials: a methodological systematic review. PLoS One.

[130] Dimairo M. The utility of adaptive designs in publicly funded confirmatory trials. 2016. http://etheses.whiterose.ac.uk/13981. Accessed 7 Jul 2017.

[131] Nie L, Rubin EH, Mehrotra N, Pinheiro J, Fernandes LL, Roy A (2016). Rendering the 3 + 3 design to rest: more efficient approaches to oncology dose-finding trials in the era of targeted therapy. Clin Cancer Res.

[132] Adaptive Designs Working Group of the MRC Network of Hubs for Trials Methodology Research. A quick guide why not to use A+B designs. 2016. https://www.methodologyhubs.mrc.ac.uk/files/6814/6253/2385/A_quick_guide_why_not_to_use_AB_designs.pdf. Accessed 7 Jul 2017.

[133] Cuffe RL, Lawrence D, Stone A, Vandemeulebroecke M (2014). When is a seamless study desirable? Case studies from different pharmaceutical sponsors. Pharm Stat.

[134] Jaki T (2015). Multi-arm clinical trials with treatment selection: what can be gained and at what price?. Clin Investig.

[135] Coffey CS, Kairalla JA (2008). Adaptive clinical trials: progress and challenges. Drugs R D.

[136] Cole M, Stocken D, Yap C. A pragmatic approach to the design and calibration of a Bayesian CRM dose finding trial. Trials. 2015;16 Suppl2:P210.

[137] Yap C, Billingham LJ, Cheung YK, Craddock C, O’Quigley J (2017). Dose transition pathways: the missing link between complex dose-finding designs and simple decision making. Clin Cancer Res.

[138] Yap C, Craddock C, Collins G, Khan J, Siddique S, Billingham L. Implementation of adaptive dose-finding designs in two early phase haematological trials: clinical, operational, and methodological challenges. Trials. 2013;14 Suppl 1:O75.

[139] Fisher AJ, Yonan N, Mascaro J, Marczin N, Tsui S, Simon A, et al. A study of donor ex-vivo lung perfusion in UK lung transplantation (DEVELOP-UK). J Heart Lung Transplant. 2016;35 Suppl 4:S80.

[140] Sydes MR, Parmar MKB, Mason MD, Clarke NW, Amos C, Anderson J (2012). Flexible trial design in practice—stopping arms for lack-of-benefit and adding research arms mid-trial in STAMPEDE: a multi-arm multi-stage randomized controlled trial. Trials.

[141] Gaunt P, Mehanna H, Yap C. The design of a multi-arm multi-stage (MAMS) phase III randomised controlled trial comparing alternative regimens for escalating (COMPARE) treatment of intermediate and high-risk oropharyngeal cancer with reflections on the complications of introducing a new experimental arm. Trials. 2015;16 Suppl 2:O16.

[142] Gerety EL, Lawrence EM, Wason J, Yan H, Hilborne S, Buscombe J (2015). Prospective study evaluating the relative sensitivity of 18F-NaF PET/CT for detecting skeletal metastases from renal cell carcinoma in comparison to multidetector CT and 99mTc-MDP bone scintigraphy, using an adaptive trial design. Ann Oncol.

[143] Ho TW, Pearlman E, Lewis D, Hämäläinen M, Connor K, Michelson D (2012). Efficacy and tolerability of rizatriptan in pediatric migraineurs: results from a randomized, double-blind, placebo-controlled trial using a novel adaptive enrichment design. Cephalalgia.

[144] Wang SJ, Hung HMJ (2013). Adaptive enrichment with subpopulation selection at interim: methodologies, applications and design considerations. Contemp Clin Trials.

[145] Kaplan R, Maughan T, Crook A, Fisher D, Wilson R, Brown L (2013). Evaluating many treatments and biomarkers in oncology: a new design. J Clin Oncol.

[146] Waldron-Lynch F, Kareclas P, Irons K, Walker NM, Mander A, Wicker LS (2014). Rationale and study design of the adaptive study of IL-2 dose on regulatory T cells in type 1 diabetes (DILT1D): a non-randomised, open label, adaptive dose finding trial. BMJ Open.

[147] Biankin AV, Piantadosi S, Hollingsworth SJ (2015). Patient-centric trials for therapeutic development in precision oncology. Nature.

[148] Antoniou M, Jorgensen AL, Kolamunnage-Dona R (2016). Biomarker-guided adaptive trial designs in phase II and phase III: a methodological review. PLoS One.

[149] Warner P, Weir CJ, Hansen CH, Douglas A, Madhra M, Hillier SG (2015). Low-dose dexamethasone as a treatment for women with heavy menstrual bleeding: protocol for response-adaptive randomised placebo-controlled dose-finding parallel group trial (DexFEM). BMJ Open.

[150] Fardipour P, Littman G, Burns DD, Dragalin V, Padmanabhan SK, Parke T (2009). Planning and executing response-adaptive learn-phase clinical trials: 2. case studies. Drug Inf J.

[151] Grieve AP (2017). Response-adaptive clinical trials: case studies in the medical literature. Pharm Stat.

[152] Whitehead J, Thygesen H, Jaki T, Davies S, Halford S, Turner H (2012). A novel phase I/IIa design for early phase oncology studies and its application in the evaluation of MK-0752 in pancreatic cancer. Stat Med.

[153] Khan J, Yap C, Clark R, Fenwick N, Marin D. Practical implementation of an adaptive phase I/II design in chronic myeloid leukaemia: evaluating both efficacy and toxicity using the EffTox design. Trials. 2013;14 Suppl 1:P20.

[154] Brock K, Billingham L, Copland M, Siddique S, Sirovica M, Yap C (2017). Implementing the EffTox dose-finding design in the Matchpoint trial. BMC Med Res Methodol.

